# Repelling *Aedes aegypti* mosquitoes with electric fields using insulated conductor wires

**DOI:** 10.1371/journal.pntd.0012493

**Published:** 2024-09-13

**Authors:** Ndey Bassin Jobe, Michael Erickson, Sarah E. Rydberg, Silvie Huijben, Krijn P. Paaijmans

**Affiliations:** 1 The Center for Evolution & Medicine, School of Life Sciences, Arizona State University, Tempe, Arizona, United States America; 2 Simon A. Levin Mathematical, Computational and Modeling Sciences Center, Arizona State University, Tempe, Arizona, United States America; 3 Wits Research Institute for Malaria, Faculty of Health Sciences, University of the Witwatersrand, Johannesburg, South Africa; Radboud University Nijmegen Radboud Institute for Molecular Life Sciences: Radboud Universiteit Radboud Institute for Molecular Life Sciences, NETHERLANDS, KINGDOM OF THE

## Abstract

**Background:**

The control and prevention of mosquito-borne diseases is mostly achieved with insecticides. However, their use has led to the rapid development and spread of insecticide resistance worldwide. Health experts have called for intensified efforts to find new approaches to reduce mosquito populations and human-mosquito contact. A promising new tool is the use of electrical fields (EFs), whereby mosquitoes are repelled by charged particles in their flight path. Such particles move between two or more conductors, and the use of uninsulated copper or aluminum plates as conductors has been proven to be effective at repelling mosquitoes. Here, for the first time, we assess if EFs generated using a single row of insulated conductor wires (ICWs) can also successfully repel mosquitoes, and whether mosquitoes are equally repelled at the same EF strength when the electrodes are a) orientated differently (horizontal vs. vertical placement), and b) spaced more apart.

**Methodology/Principal findings:**

Over a period of 23 hours, the number of host-seeking female *Aedes aegypti* mosquitoes that were successfully repelled by EFs, using ICWs, at EF strengths ranging from 0 kV/cm (control) to 9.15 kV/cm were quantified. Mosquitoes were released inside a 220×220×180 cm room and lured into a BG-Pro trap that was equipped with a BG-counter and baited with CO_2_ using dry ice. Mosquitoes had to pass through an EF window, that contained a single row of ICWs with alternating polarity, to reach the bait. The baseline interaction between EF strength and repellency was assessed first, after which the impact of different ICW orientations and ICW distances on repellency were determined.

Over 50% of mosquitoes were repelled at EF strengths of ≥ 3.66 kV/cm. A linear regression model showed that a vertical ICW orientation (vertical vs. horizontal) had a small but insignificant increased impact on mosquito repellency (p = 0.059), and increasing ICW distance (while maintaining the same EF strength) significantly reduced repellency (p = 0.01).

**Conclusions/Significance:**

ICWs can be used to generate EFs that partially repel host-seeking mosquitoes, which will reduce human-mosquito contact. While future studies need to assess if (i) increased repellency can be achieved, and (ii) a repellency of 50–60% is sufficient to impact disease transmission, it is encouraging that EF repellency using ICWs is higher compared to that of some spatial repellent technologies currently in development. This technology can be used in the housing improvement toolkit (i.e. preventing mosquito entry through eaves, windows, and doors). Moreover, the use of cheap, over-the-counter ICWs will mean that the technology is more accessible worldwide, and easier to manufacture and implement locally.

## Author summary

Mosquito-borne diseases cause significant public health challenges worldwide. To control and prevent the spread of these diseases, mainly insecticides are used to reduce mosquito population sizes and/or prevent human-mosquito contact. However, their excessive usage has led to the rapid development of insecticide resistance, which warrants the need for the development of new mosquito control tools. This study investigates the use of electric fields that are created between insulated conductor wires to repel *Aedes aegypti* mosquitoes. We found that electric fields can effectively repel over 50% of host-seeking female *Aedes aegypti* mosquitoes at electric field strengths of ≥ 3.66 kV/cm. There are several advantages to using this novel mosquito control tool compared to existing electric field technologies. These include the affordability and accessibility of wires, making the technology suitable for widespread implementation especially in low-middle income countries that are mostly affected by mosquito-borne diseases. This technology has the potential to reduce human-mosquito contact which is a critical factor in mosquito-borne disease transmission.

## Introduction

Mosquito-borne diseases (MBDs) cause a major threat to global human health [1]. Malaria is one of the deadliest MBDs, accounting for an estimated 249 million cases and 608,000 deaths in 2022 alone, globally [2]. In addition, other MBDs, such as Zika and dengue, have imposed a significant burden to human health. For instance, there were 500,000 suspected cases of Zika in the Americas in 2016 [1]. According to the WHO, the reported cases of dengue increased from about half a million in 2000 to 5.2 million in 2019 [3].

Insecticides (used in e.g. bednets, indoor residual spraying and space spraying) have predominantly been used to control and prevent existing and (re)emerging MBDs [4–7]. Those interventions are effective [7–10] as they reduce mosquito population sizes and/or prevent human-vector contact. However, their excessive usage raises concerns for our environment (e.g. non-target organisms) [11–13] and health [14,15], and has led to the rapid development and spread of insecticide resistance [16,17]. As a result, intensified efforts are needed to create novel vector control tools [18–20].

One of the new tools in development is the use of electrical fields (EFs) to control insects [21–24]. EFs are created by charges, whereby a charged particle moves through space from a positively (or negatively) charged electrode conductor to a grounded electrode conductor. Although there is little known about how EFs exactly affect insects [25], it has been shown that EFs can repel insects [21,23,24]. This is most likely a result of charged particles deflecting the antennae [26] or mechanosensory hairs on an insect’s body [27], which results in a neural response, but understanding and leveraging the electroreceptive neurobiology of insects remains an important area of future research [25].

To-date, the efficacy of EFs in repelling *Ae*. *aegypti* mosquitoes has been assessed using two different conductors: copper plates and aluminum blinds. In that setup, a single row of conductors with alternating polarity successfully repelled over 90% of *Ae*. *aegypti* mosquitoes at EF strengths of 1.25 kV/cm (i.e. 2.5 kV applied to positively-charged conductors spaced 2 cm from the adjacent grounded conductors) and 1.5 kV/cm for copper plates and aluminum blinds, respectively [21]. ICWs have also been used in the past to effectively repel *Aedes albopictus* mosquitoes, whereby all mosquitoes were repelled at an EF strength of 1.67 kV/cm [23]. However, in their setup, the EF was created between a row of parallel ICWs that were all negatively charged and a single earthed stainless-steel mesh plate that was placed in parallel of the ICWs [23]. In other words, they tested two rows of conductors, which means that a mosquito that approaches the electric field experiences charged particles that move in its direction (i.e. from the negative ICWs to the earthed plate, thus towards the mosquito).

The EF technology is currently being integrated into outdoor aluminum window blinds, a product that will be marketed to high-end consumers due to its price. To make the technology more accessible (e.g. across socioeconomic status, in low-middle income countries and/or underserved communities that are typically hit hardest by MBDs [28,29])) and wider implementable (protecting a wide range of mosquito-accessible spaces), the repellency effect of EFs that are created by other, low cost, types of conductors such as insulated conductor wires (or ICWs) need to be evaluated. The additional benefit of using ICWs is that it is easier to replicate experiments by other researchers, allowing for reproducibility and comparability of results. Here we assess the repellency of EFs that are created by a single row of insulated conductor wires with alternating polarity, whereby the charged particles move more perpendicular (i.e. from a positive ICW to the adjacent earthed ICWs next to it) in relation to an approaching mosquito. In addition, we address two important questions related to the future configuration of EF setups: Are mosquitoes equally repelled at the same EF strength when the electrodes are a) orientated differently (horizontal vs. vertical placement), and b) spaced more apart.

## Methods

### Mosquito rearing and preparation

Four-to-six days old non-blood fed adult female *Aedes aegypti* MC1 mosquitoes (isolated from Maricopa County, Arizona, in 2018 and reared in our lab since 2019) were used for the entirety of this study. The mosquitoes were reared at 27°C, 80% RH and a 12h photoperiod in walk-in climate chambers (Model No. ENV-G1HD-8X12, Darwin Chambers, Missouri, USA) following procedures described elsewhere [30]. Adults had access to 10% sugar water (refined household sugar), which was removed 24h before the start of each experimental run. Hundred host-seeking female mosquitoes (attracted to a human hand outside the cage) were selected daily for each experimental run.

### Experimental set-up

**Fig 1:**
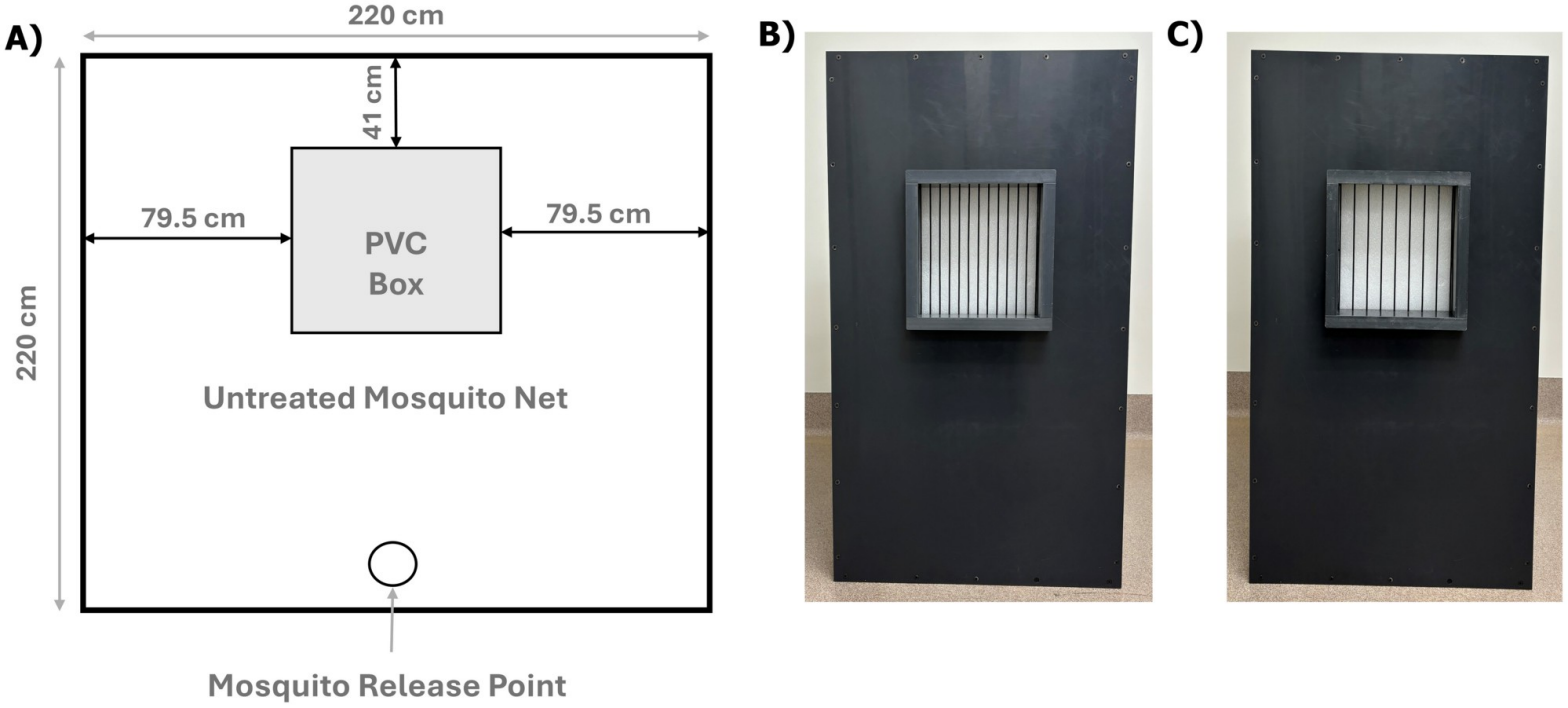
A) Schematic drawing of the top view of our experimental setup, including the position of the PVC Box with EF window and mosquito release point, and images of the front view of the PVC Box with the EF window with ICWs spaced B) 1.64 cm, and C) 2.64 cm apart.

Mosquitoes were released daily at 11:00 h inside a large untreated mosquito net enclosure (*l×w×h*: 220×220×180 cm, Model No. MPRO101, Mekkapro, China) and lured into a BG-Pro trap (Biogents AG, Regensburg, Germany) that was equipped with a BG-counter (Biogents AG, Regensburg, Germany) using CO_2_ as bait (dry ice, 4 lbs. per experimental run). The BG-counter recorded the exact time (hours, minutes, seconds) when a mosquito entered the trap, and this data was wirelessly transmitted and stored to a cloud server. The trap and dry ice were placed inside a PVC box (*l×w×h*: 61×61×102 cm) that had one opening for an ‘EF window’ (see ‘Electric field (EF) window’). This opening was located 21.7 cm from the top of the box, 50.8 cm from the bottom of the box, and equidistant from the sides (15.75 cm). Mosquitoes had to pass through this window to reach the bait/trap. Placement of the box in relation to the mosquito net and mosquito release point is shown in Fig 1.

The room was programmed on a 12h photoperiod (i.e., 07:00 h lights on; 19:00 h lights off). Room temperature (°C) and relative humidity (RH) were not regulated but monitored with a datalogger (Model No. OM-92-NIST, accuracy: ±0.3°C and ±3% RH, Omega, Connecticut, USA) during each experimental run (Table 1). Every morning at 10:00 h (i.e. 23h after release), all mosquitoes were recaptured, and it was noted if they were collected i) outside the PVC box, ii) inside the PVC box but not in the trap, or iii) in the catch bag of the trap.

**Table 1:** Mean temperature and relative humidity (± standard deviation) in the flight room for each of the three separate experiments.

	Mean Temperature (°C) ± SD	Mean Relative Humidity (%) ± SD
EXP 1	21.9 ± 0.1	54.2 ± 2.8
EXP 2	21.8 ± 0.2	48.4 ± 9.2
EXP 3	21.4 ± 0.1	30.8 ± 7.9

### Electric field (EF) window

Each EF window frame (outer dimension 29.5×29.5 cm; inner dimensions 24.4×24.4 cm) consisted of a single row of parallel-placed ICWs (insulation: polyvinyl chloride, 14 AWG, 0.36 cm diameter, rated for 600V AC) with alternating polarity (starting and ending with a positively-charged ICW). Wires were either placed 2 or 3 cm apart (depending on the experiment, see below) from the center of the wires, which results in distances between ICWs of 1.64 or 2.64 cm, respectively. The latter distance is important, as the strength of an EF (expressed as kV/cm) was calculated for the distance between the circumference of a positively-charged and the circumference of a grounded electrode conductor in each experimental run. EFs were generated using a High Voltage Power Supply unit with a positive polarity (Model No. 73530, output range 0-35kV, 0.85mA, and 30W, Genvolt, Shropshire, UK), and measured using a high voltage probe (Model No. HVP40, direct current accuracy ± 1% to 20KV, RSR Electronics, New Jersey, USA).

### Experimental design

#### Experiment 1) Assessing the voltage-repellency interaction of EFs using vertically-placed ICWs

ICWs in the EF window were placed vertically, and the distance between the outsides of the ICWs was 1.64 cm (Fig 1B). The repellency of *Ae*. *aegypti* mosquitoes was assessed at 0 (control), 3, 6, 9, 12, and 15 kV, resulting in EF strengths of 0, 1.83, 3.66, 5.49, 7.32, and 9.15 kV/cm, respectively. In a subsequent experiment, repellency was monitored at 0, 1, 2, and 9 kV (or 0, 0.61, 1.22 and 5.49 kV/cm) to obtain additional information for the region between zero and the maximum repellency. For both experiments, there were three replicate studies. During each replicate study, all EF strengths were tested in random order.

#### Experiment 2) Assessing the effects of ICW orientation on *Ae*. *aegypti* repellency

The same EF window was used and placed inside the PVC box in either a vertical (as experiment 1) or horizontal position. EF strengths of 0, 0.61, 1.83, and 3.66 kV/cm were applied. The ICW orientation and voltages were tested in random order and the full experiment was replicated three times.

#### Experiment 3) Assessing the effects of ICW distance on *Ae*. *aegypti* repellency

An EF window with wires spaced 1.64 cm apart (experiments 1 and 2) was compared to an identical window with wires spaced 2.64 cm apart (Fig 1C). EF strengths of 0, 0.61, 1.83, and 3.66 kV/cm were applied. The ICW distances and voltages were tested in random order and the full experiment was replicated three times.

### Data analysis

To calculate repellency and thus correct for the fact that not all mosquitoes passed through the window in the control treatment (i.e. 0 kV/cm), Abbott’s formula [31] was applied at each EF strength by:

$$\frac{\left({N_{net}}^{kV}\;–\text{mean}\;{N_{net}}^{0kV}\;\right)}{100-\text{mean}\;{N_{net}}^{0kV}}*100$$

with *N*_*net*_^*kV*^ being the number of mosquitoes that did not pass through the EF window at each EF strength, and mean *N*_*net*_^0*kV*^ being the mean number of mosquitoes that did not pass through the EF window in the control (0 kV) treatments.

For experiments 2 and 3, a generalized linear model was used to determine the effect of EF strength and ICW orientation (experiment 2) or ICW distance (experiment 3) and their interaction on mosquito repellency. Interaction terms that were insignificant were subsequently removed from the model. This was followed by a two-sample t-test at each individual EF strength to determine differences in repellency between the treatments (ICW orientation or ICW distance).

The time of entry of each *Ae*. *aegypti* mosquito (as recorded by the BG-counter) was rounded to the nearest 15 minutes for each experimental run. Mosquitoes that passed the EF window but were not captured in the trap were not included in the time of entry analysis (S1 File). Due to technical issues, data for two experimental observations in experiment 3 are missing (0 kV/cm at ICW distance of 1.64 cm, 1.83 kV/cm at ICW distance of 2.64 cm). All data analyses and visualizations were performed using R version 4.3.2. [32].

## Results

### Experiment 1) Assessing the voltage-repellency interaction of EFs using vertically-placed ICWs

**Fig 2:**
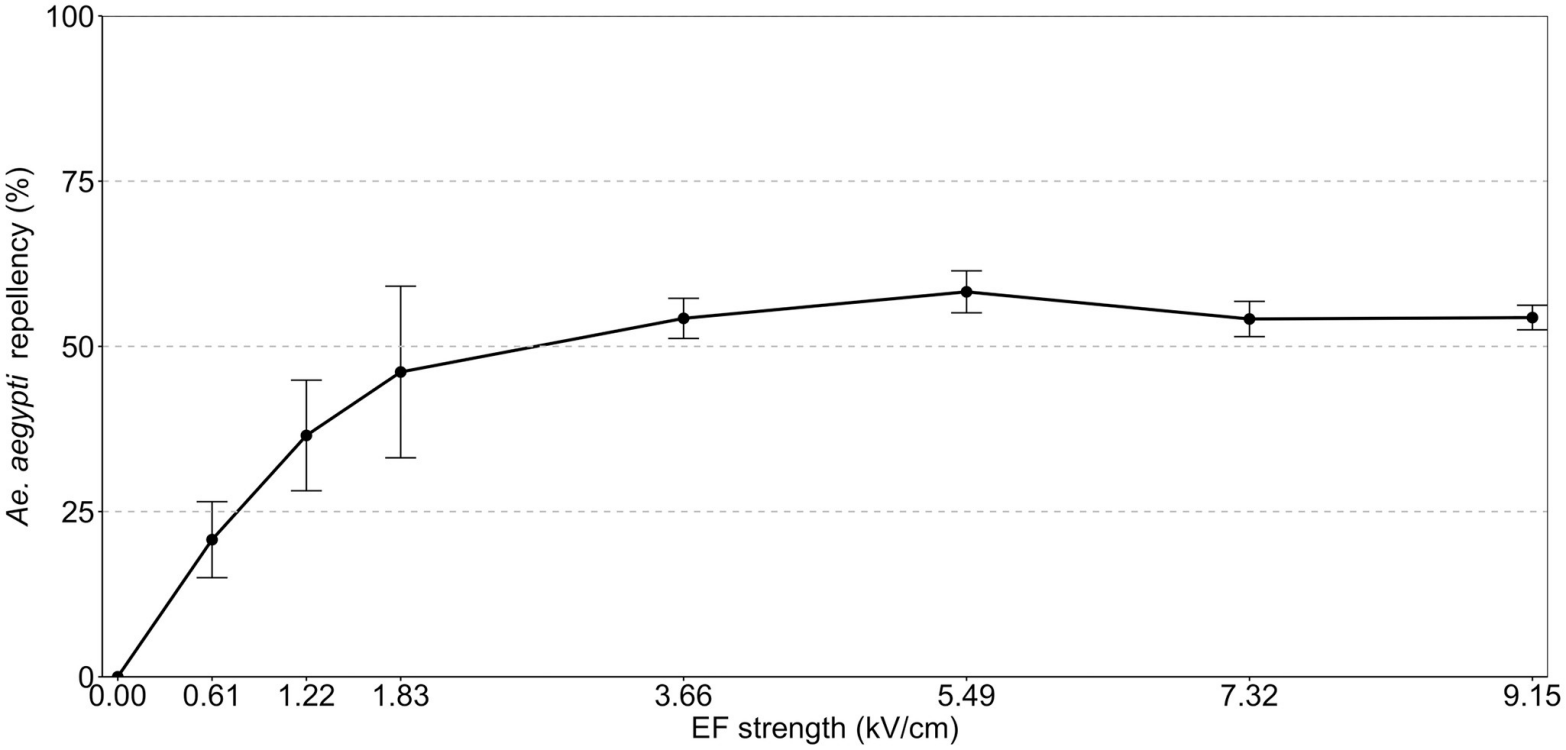
Voltage-repellency interaction of EFs created with vertically-placed ICWs. The x-axis shows the different tested electric field strengths (kV/cm), and the y-axis shows the Abbott-corrected mean percent repellency of *Ae*. *aegypti* females. The bars represent the standard error of the means.

*Ae*. *aegypti* mosquitoes were significantly repelled by electrical fields. Repellency increased as EF strength increased but leveled off at EF strengths of ≥ 3.66 kV/cm to 54.1%–58.3% repellency (Fig 2).

### Experiment 2) Assessing the effects of ICW orientation on *Ae*. *aegypti* repellency

**Fig 3:**
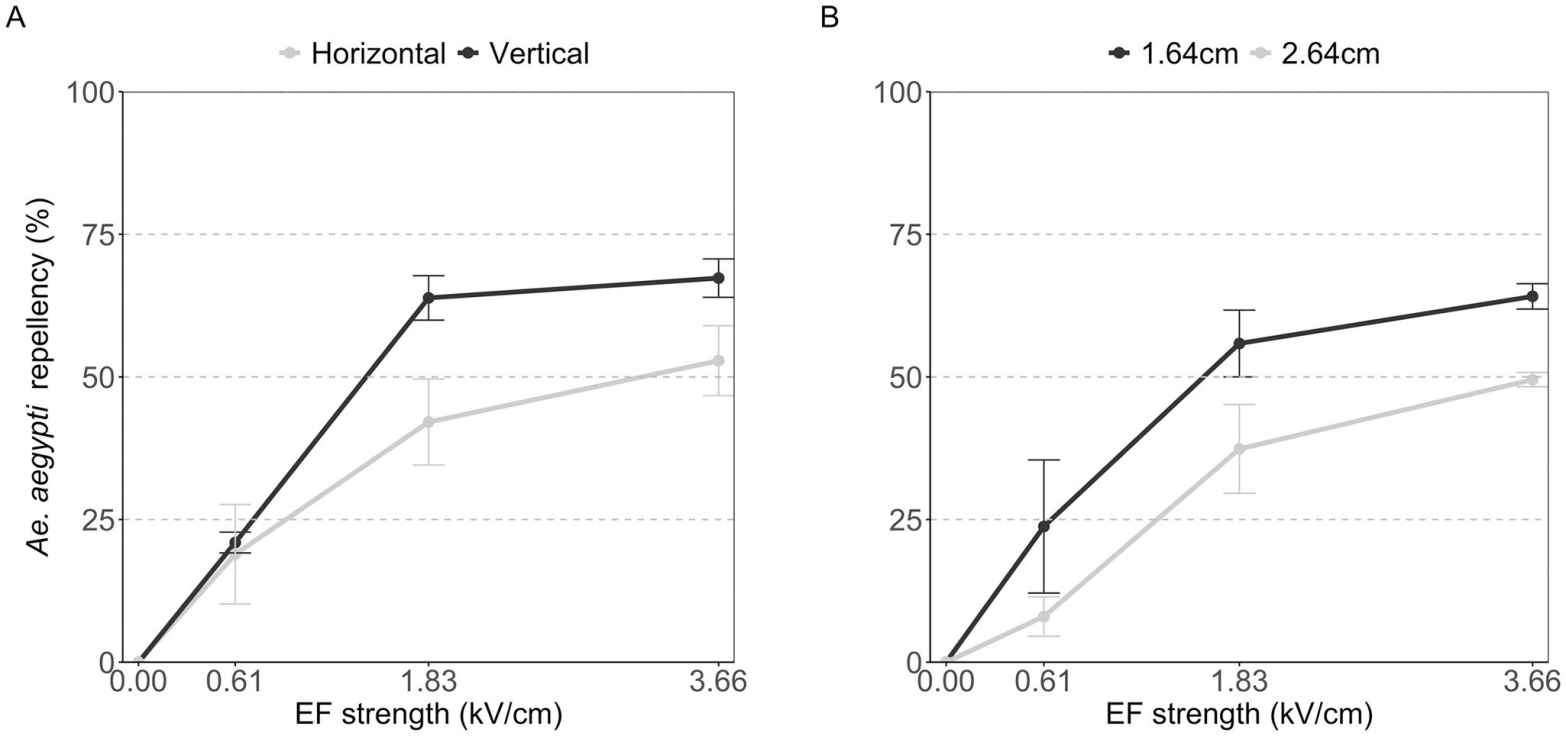
*Aedes aegypti* repellency using electric fields with (A) different ICW orientations (i.e. vertical (black line) and horizontal (grey line)), and (B) different distances between the ICW (i.e. 1.64 cm (black line) and 2.64 cm (grey line)). The x-axis shows the different tested electric field strengths (kV/cm), and the y-axis shows the Abbott-corrected mean percent repellency of *Ae*. *aegypti* females. The bars represent the standard error of the means.

At increased EF strength, there appeared to be a greater repellency at vertical placement of the wires than at horizontal placement (Fig 3A). However, this effect was not significant (EF strength: p < 0.001, orientation: p = 0.059). Individual comparison at each individual EF strength (0.61, 1.83 and 3.66 kV/cm) similarly did not show significant differences between both orientations (p = 0.84, 0.08 and 0.13, respectively).

### Experiment 3) Assessing the effects of ICW distance on *Ae*. *aegypti* repellency

A significant decrease in repellency was observed with increased distance between the wires across all voltages (EF strength: p < 0.001, distance: p = 0.01, Fig 3B). Overall, increasing the distance between conductors from 1.64 cm to 2.64 cm—but keeping the EF strength constant—was associated with a 16% decrease in mosquito repellency.

### *Aedes aegypti* time of entry

**Fig 4:**
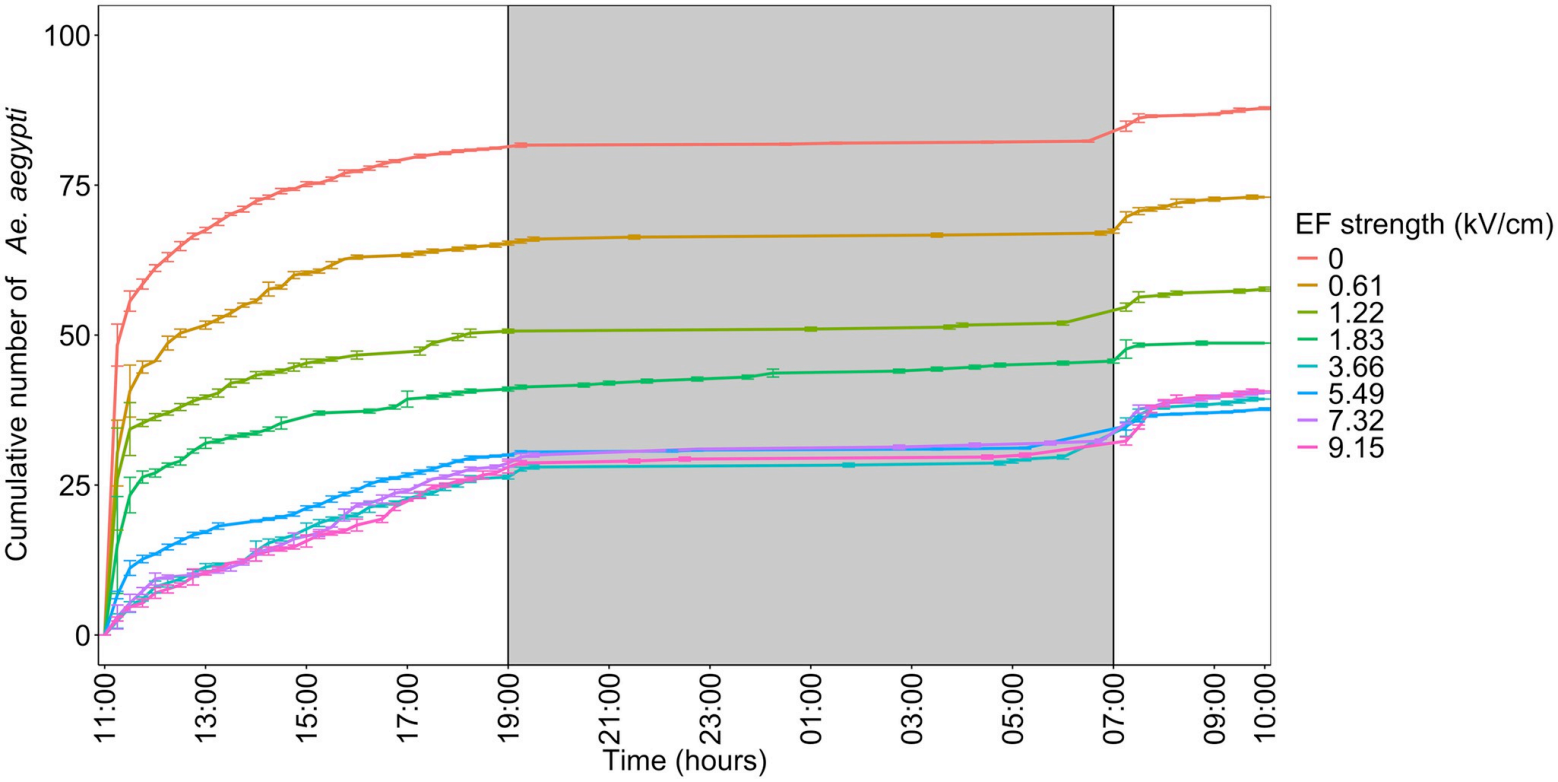
Time of trap entry of *Ae*. *aegypti* during experiment 1. The x-axis shows the time of entry of *Ae*. *aegypti* (from their release at 11:00 h until the end of the experiment at 10:00 h the next day). The y-axis shows the cumulative mean number of *Ae*. *aegypti* females that passed through the EF window and were captured in the BG-pro trap. The bars represent the standard error of the means. The shaded area indicates nighttime. Note that the final number of *Ae*. *aegypti* that passed through the EF window does not necessarily correspond to these data, as some mosquitoes were collected inside the PVC box, and not captured in the mosquito trap.

Most *Ae*. *aegypti* were captured during daylight hours, that is, between their time of release (11:00 h) and the time the room lights switched off (19:00 h), followed by the period between when the lights turned back on (7:00 h) and the end of the experiment (10:00 h). Fewer mosquitoes were collected during nighttime (19:00 h—7:00 h). This pattern was consistent between EF strengths tested, and ICW orientation and distance. To illustrate, in experiment 1, across all EF strengths tested, 67–92%, 0–6%, and 7–29% of *Ae*. *aegypti* mosquitoes were captured between 11:00 h -19:00 h, 19:00 h—07:00 h and 07:00 h—10:00 h, respectively (Fig 4; S1A Table). Data from the other experiments is similar (S1A and S1B Fig and S1A, S1B and S1C Table).

## Discussion

This study introduces a novel approach using insulated conductor wires (ICWs) to generate electric fields that prevent host-seeking female *Aedes aegypti* from entering spaces. The ICWs used here generated EFs that repelled over 50% of mosquitoes at ≥3.66kV/cm. At similar EF strengths, there was a potential increased repellency with vertically placed wires (p = 0.059) that requires further investigation. A clear increase in repellency was observed when the wires were placed closer together within the window (p = 0.01).

Other studies have shown higher repellency of mosquitoes using electric fields [21,23]. In a study by Gordon and colleagues, parallel-placed copper plates and aluminum blinds repelled over 90% of *Ae*. *aegypti* at EF strengths of 1.25 kV/cm and 1.5 kV/cm, respectively [21]. Matsuda and colleagues were able to repel 100% of *Ae*. *albopictus* mosquitoes at an EF strength of 1.67 kV/cm using a row of negatively charged parallel ICWs and an earthed stainless-steel mesh plate on one side of the ICWs [23]. The higher repellency achieved by Gordon and colleagues is most likely due to differences in the EF depth (i.e. 1 cm for their copper plates and 2 cm for their aluminum blinds *vs*. 0.36 cm in our setup), which could impact mosquito EF perception. While the EF depth in Matsuda’s study was more comparable to ours (0.3 cm *vs*. 0.36 cm), it is important to note that their setup was different from ours. They used two rows of conductors (ICWs and stainless-steel mesh plate) instead of one row of conductor (ICWs alone), and all ICWs were charged similarly and negatively (vs. alternating ICW polarity in our setup, powered using a positive voltage generator). Unfortunately, they did not describe important experimental details, such as (i) the rearing conditions, sex and age of the mosquitoes tested, (ii) time of each test/experimental run, and (iii) the lure used, if any, to attract the mosquitoes towards the EF screen, which makes it impossible to fully interpret the data.

Luan and colleagues were able to prevent 100% of *Ae*. *aegypti* mosquitoes from blood feeding using a very low voltage (15V). They used a cloth (3-D textile) that was composed of dual layers of conductive fabrics that were electrically insulated by a fiberglass mesh fabric in the middle [33]. However, it is clear from our experiments and other work [21,23] that the insect barrier approach we are developing will require higher voltages. Both ours and the aforementioned studies do highlight that future studies should assess the impact of different ICW configurations, shapes, and gauges, and different voltage generators (positive or negative), and ideally use similar experimental protocols that will enable us to compare insect repellency results.

It appeared that there was a greater repellency at vertical placement of the wires than at horizontal placement (Fig 3A). While this effect was not significant, it is likely that there is a true effect of EF orientation on repellency as mosquitoes are known to respond differently to the orientation of flight openings. *Anopheles gambiae* showed a higher flight activity preference towards horizontally-oriented compared to vertically-oriented holes in bednets [34]. This observation was attributed to the “edge effect” in vertically-oriented holes, whereby mosquitoes collided with the edges of the holes when they extend their wings and legs during flight. Here we indeed observed increased repellency in vertically-oriented electric fields than horizontally-oriented fields that goes beyond a physical effect, since repellency is corrected for by repellency in the absence of an electric field. Likely due to the low sampling size at each EF strength and the non-linearity of the EF strength-repellency relationship in the EF range measured, these differences were not significant. Increasing the sample size and including tests at higher EF strengths in future studies are needed to better understand the impact of EF orientation on mosquito repellency.

An increase in the distance between conductors, while keeping the EF strength constant, resulted in decreased mosquito repellency. The EF strength between two conductors is directly proportional to the charge and inversely proportional to the distance between the surfaces [21,35]. In other words, applying 1 kV to conductors spaced 1.64 cm apart, and 1.61 kV when they are 2.64 cm apart results in the same EF strength of 0.61 kV/cm. Further tests across a range of distances are needed to establish the relationship between distance and repellency to determine the optimal distance and EF strength for maximum mosquito repellency.

Regarding *Ae*. *aegypti* host-seeking behaviors, mosquitoes exhibited reduced activity during the nighttime hours, when lights were off in the room. Most mosquitoes that passed the EF window were caught before 19:00 h, followed by mosquitoes passing through during the morning hours (7:00 h—10:00 h). These findings are consistent with their known natural behavior, as *Ae*. *aegypti* is a typical daytime (diurnal) biter [36–38]. Understanding this behavior is important, as in theory the EF technology can be switched on during periods of time when mosquitoes are active, and switched off when mosquitoes are not active, which could benefit energy costs and product lifespan.

There are a few limitations to the present study. First, the ICWs used for this study were rated for 600V. As voltages higher than 600V may be needed to effectively repel mosquitoes (depending on the distance between electrodes), this raises concerns for end-user safety of the product in its current setup. Future tests should be conducted with high-voltage rated wires (i.e. ICWs that are designed to safely conduct e.g. 5 kV or 10 kV) that are safe to touch. Alternatively, conductors can be embedded between two layers that are safe to touch (e.g., plastic mesh plates on either side), or insulated with protector sleeves. In addition, a large resistor of ≥1 mega-ohm can be integrated with a series of the output making even the use of non-insulated conductors safe (limiting the flow of current through the human body to 4 mA) in case of accidental touching [21]. It is also worth noting that Biogents (Regensburg, Germany) have developed an HV generator prototype that can be supplied with 12 V DC power from a variety of power sources, including primary and secondary batteries (Lead Acid, Lithium-ion), plug-in power supplies using AC to DC converters or solar panels [21]. They are currently evaluating the implementation of pulsed electric fields that would reduce operating costs and extend battery life.

Second, our study was conducted in a laboratory setting, using a laboratory-reared strain of a single mosquito species. As laboratory findings do not always translate to field observations (e.g. [39,40]), field trials with the EF technology are necessary. Considering multiple mosquito genera (e.g. *Anopheles* species that vector malaria, other *Aedes* species that vector Zika and dengue, and *Culex* species that vector West Nile virus) will provide valuable insights into the potential impact of using the EF technology to control a broad range of MBDs. Semi-field and field testing will also allow us to assess the impact of different climatic conditions on EF repellency, as external factors such as temperature and humidity affect both mosquito behavior [41,42] as well as the EF itself [43,44]. Impact assessments of the EF technology should ideally not be restrained to entomological outcomes but include evaluating the reduction in disease burden. A modeling framework may also allow us to estimate the community level impact (e.g. [45]) of the EF technology across different geographical locations and at different repellency efficacies.

Our findings demonstrate that *Ae*. *aegypti* mosquitoes can be effectively repelled by EFs that are created using ICWs. Although repellency intuitively may seem low (50–60% in this study compared to >90% in a previous study [21]), it could still significantly impact disease transmission. The repellency values here are between those reported for spatial repellents (SR), which showed low (e.g. 16.4% [46]) to high (e.g. 70% [47]) reductions in malaria mosquito-human contact. Furthermore, a model study predicted that spatial repellent emanator devices can reduce the malaria burden when added to long-lasting insecticidal nets, even though the reduction in mosquito-human contact went from 76% (right beneath the SR emanator device, at zero distance) to 50% (2.5 m away from the SR emanator device, a more realistic distance in households) [48]. One interesting hypothesis that has been raised is whether incomplete repellency, when combined with effective indoor vector-control tools, could lead to the evolution of increased repellency in mosquito populations [49]. The rationale is that the mosquitoes that are not repelled are eventually killed by a second intervention, which could lead to a selection for those mosquitoes that are repelled over time. On the flipside, given that the EF does not repel all mosquitoes, it would be worthwhile to investigate whether mosquitoes can evolve to resist these electric fields.

In conclusion, ICWs are inexpensive and widely available in many areas, and as such, the EF technology presented here could be particularly beneficial to low-middle income countries and underserved communities that are typically hit hardest by MBDs [28,29]. However, the feasibility in relation to installation (e.g. expertise required), obtaining a power source that generates and maintains the required EF strength for repellency and electrical safety should be further studied. Overall, the role of ICWs in EF technologies for vector control should be explored, as it can be an accessible tool in e.g. the housing improvement toolkit, as the conductors can be installed in mosquito entry points (eaves, windows, and/or doors), preventing mosquito entry similar to current technologies such as eave tubes [50], transfluthrin-treated eave ribbons [51], screened windows [52], and doors [53]. Investigating these aspects will not only refine the efficacy of EF technologies but also pave the way for a more equitable and sustainable approach to combating mosquito-borne diseases in resource-limited settings.

## Supporting information

S1 Fig **A.** Time of trap entry of *Ae*. *aegypti* during experiment 2. **B.** Time of trap entry of *Ae*. *aegypti* during experiment 3. The x-axis shows the time of entry of *Ae*. *aegypti* (from their release at 11:00 h until the end of the experiment at 10:00 h). The y-axis shows the cumulative mean number of *Ae*. *aegypti* females that passed through the EF window and were captured in the BG-pro trap. The bars represent the standard error of the means. Note that the final number of *Ae*. *aegypti* that passed through the EF window does not necessarily correspond to the data in this file, as some mosquitoes were collected inside the PVC box, and not captured in the mosquito trap.
(DOCX)

S1 Table The percentage of *Ae*. *aegypti* females that passed through the EF window and were captured in the BG-pro trap. Each column is expressed out of all *Ae*. *aegypti* females captured by 10:00 h the next day (i.e. 23 hours later). **A**. Experiment 1, **B**. Experiment 2, **C**. Experiment 3.
(DOCX)

S1 File Electric field—Mosquito repellency dataset, grouped by experiment (tabs). (XLSX)

S2 File Temperature and humidity dataset, grouped by experiment (tabs). (XLSX)

S3 File Mosquito trap entry time dataset for experiment 1, grouped by electric field strength tested (tabs). Every two columns show data from a single experimental run.
(XLSX)

S4 File Mosquito trap entry time dataset for experiment 2, grouped by electric field strength tested (tabs). Every two columns show data from a single experimental run.
(XLSX)

S5 File Mosquito trap entry time dataset for experiment 3, grouped by electric field strength tested (tabs). Every two columns show data from a single experimental run.
(XLSX)
