# Supplementary material for: Repelling Aedes aegypti mosquitoes with electric fields using insulated conductor wires
Source: PLoS Negl Trop Dis. 2024 Sep 13;18(9):e0012493. doi: 10.1371/journal.pntd.0012493 (PMC11424001; doi:10.1371/journal.pntd.0012493)
Supplement: S1 Fig — A. Time of trap entry of Ae. aegypti during experiment 2. B. Time of trap entry of Ae. aegypti during experiment 3. The x-axis shows the time of entry of Ae. aegypti (from their release at 11:00 h until the end of the experiment at 10:00 h). The y-axis shows the cumulative mean number of Ae. aegypti females that passed through the EF window and were captured in the BG-pro trap. The bars represent the standard error of the means. Note that the final number of Ae. aegypti that passed through the EF window does not necessarily correspond to the data in this file, as some mosquitoes were collected inside the PVC box, and not captured in the mosquito trap. (DOCX) [file pntd.0012493.s001.docx]

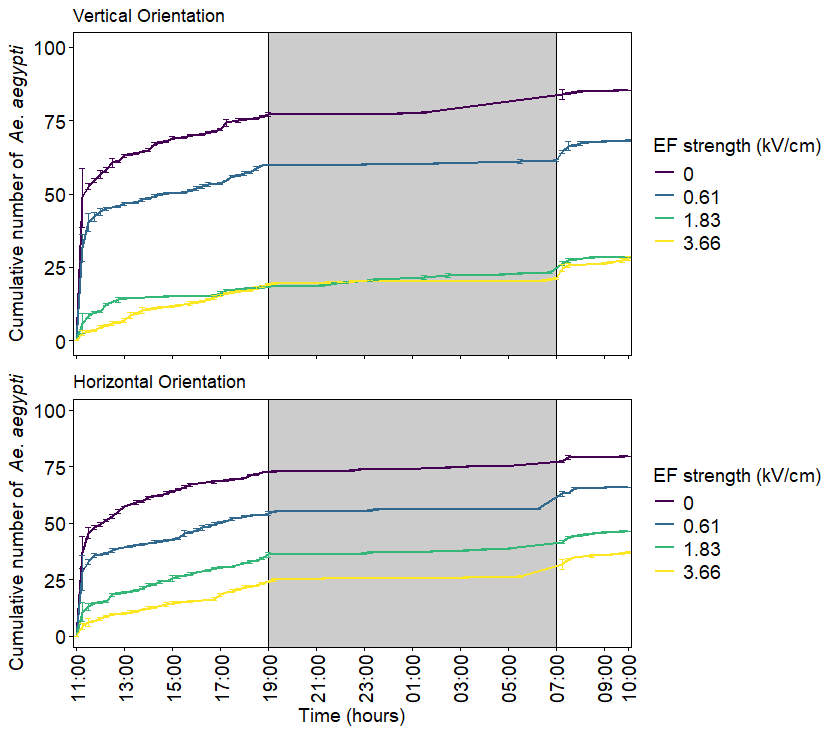


**S1A Fig.** Time of trap entry of *Ae. aegypti* during experiment 2. The x-axis shows the time of entry of *Ae. aegypti* (from their release at 11:00 h until the end of the experiment at 10:00 h the next day). The y-axis shows the cumulative mean number of *Ae. aegypti* females that passed through the EF window and were captured in the BG-pro trap. The bars represent the standard error of the means. The shaded area indicates nighttime. Note that the final number of *Ae. aegypti* that passed through the EF window does not necessarily correspond to these data, as some mosquitoes were collected inside the PVC box, and not captured in the mosquito trap.

**
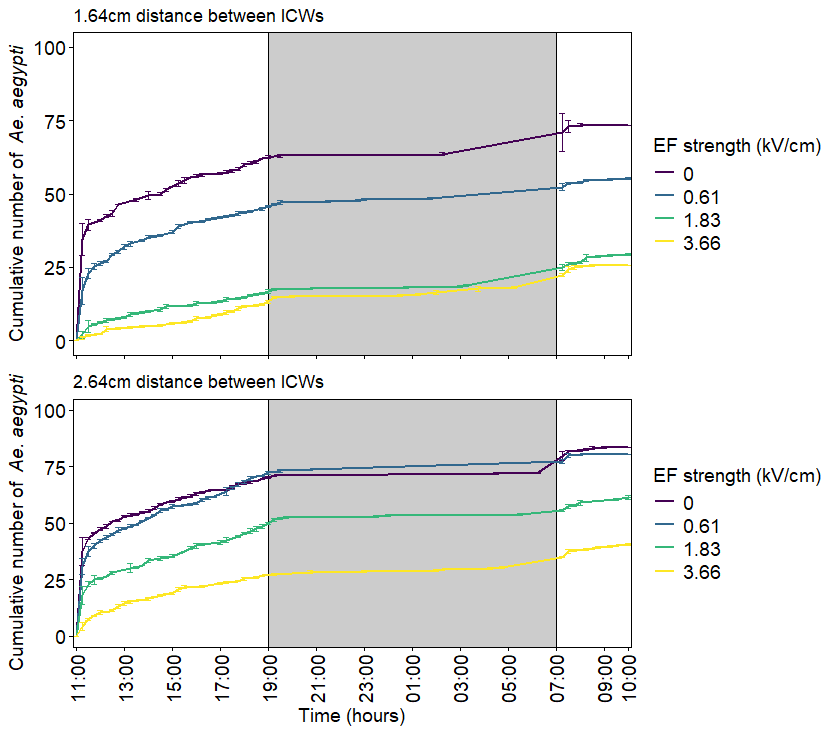
**

**S1B Fig.** Time of trap entry of *Ae. aegypti* during experiment 3. The x-axis shows the time of entry of *Ae. aegypti* (from their release at 11:00 h until the end of the experiment at 10:00 h the next day). The y-axis shows the cumulative mean number of *Ae. aegypti* females that passed through the EF window and were captured in the BG-pro trap. The bars represent the standard error of the means. The shaded area indicates nighttime. Note that the final number of *Ae. aegypti* that passed through the EF window does not necessarily correspond to these data, as some mosquitoes were collected inside the PVC box, and not captured in the mosquito trap.
