# Supplementary material for: Repelling Aedes aegypti mosquitoes with electric fields using insulated conductor wires
Source: PLoS Negl Trop Dis. 2024 Sep 13;18(9):e0012493. doi: 10.1371/journal.pntd.0012493 (PMC11424001; doi:10.1371/journal.pntd.0012493)
Supplement: S1 Table — Each column is expressed out of all Ae. aegypti females captured by 10:00 h the next day (i.e. 23 hours later). A. Experiment 1, B. Experiment 2, C. Experiment 3. (DOCX) [file pntd.0012493.s002.docx]

**S1A Table. The percentage of *Ae. aegypti* females that passed through the EF window and were captured in the BG-pro trap in experiment 1.** Each column is expressed out of all *Ae. aegypti* females captured by 10:00 h the next day (i.e. 23 hours later).

| **Voltage (kV/cm)** | **11:00-19:00 h** | **19:00-07:00 h** | **07:00-10:00 h** |
| --- | --- | --- | --- |
| **0** | 92.4 | 0.6 | 7.0 |
| **0.61** | 89.5 | 1.4 | 9.1 |
| **1.22** | 87.9 | 0 | 12.1 |
| **1.83** | 84.2 | 4.1 | 11.6 |
| **3.66** | 66.9 | 4.2 | 28.8 |
| **5.49** | 79.6 | 2.2 | 18.1 |
| **7.32** | 71.1 | 5.8 | 23.1 |
| **9.15** | 68.9 | 3.3 | 27.9 |

**S1B Table. The percentage of *Ae. aegypti* females that passed through the EF window and were captured in the BG-pro trap in experiment 2.** Each column is expressed out of all *Ae. aegypti* females captured by 10:00 h the next day (i.e. 23 hours later).

| **Voltage (kV/cm)** | **11:00-19:00 h** | **19:00-07:00 h** | **07:00-10:00 h** |
| --- | --- | --- | --- |
| Vertical orientation | | | |
| **0** | 90.2 | 0 | 9.8 |
| **0.61** | 87.3 | 0.5 | 12.2 |
| **1.83** | 65.9 | 8.2 | 25.9 |
| **3.66** | 69.0 | 3.6 | 27.4 |
| Horizontal orientation | | | |
| **0** | 91.2 | 1.3 | 7.5 |
| **0.61** | 82.3 | 2.5 | 15.2 |
| **1.83** | 77.7 | 2.2 | 20.1 |
| **3.66** | 64.9 | 4.5 | 30.6 |

**S1C Table. The percentage of *Ae. aegypti* females that passed through the EF window and were captured in the BG-pro trap in experiment 3.** Each column is expressed out of all *Ae. aegypti* females captured by 10:00 h the next day (i.e. 23 hours later).

| **Voltage (kV/cm)** | **11:00-19:00 h** | **19:00-07:00 h** | **07:00-10:00 h** |
| --- | --- | --- | --- |
| 1.64 cm distance | | | |
| **0** | 85.0 | 0.7 | 14.3 |
| **0.61** | 82.5 | 4.22 | 13.3 |
| **1.83** | 56.8 | 4.5 | 38.6 |
| **3.66** | 51.9 | 6.5 | 41.6 |
| 2.64 cm distance | | | |
| **0** | 84.1 | 1.2 | 14.7 |
| **0.61** | 89.7 | 1.7 | 8.7 |
| **1.83** | 81.3 | 4.9 | 13.8 |
| **3.66** | 65.6 | 4.9 | 29.5 |
